# Thrombin-derived C-terminal fragments aggregate and scavenge bacteria and their proinflammatory products

**DOI:** 10.1074/jbc.RA120.012741

**Published:** 2020-02-07

**Authors:** Jitka Petrlova, Ganna Petruk, Roland G. Huber, Eilish W. McBurnie, Mariena J. A. van der Plas, Peter J. Bond, Manoj Puthia, Artur Schmidtchen

**Affiliations:** ‡ Department of Clinical Sciences, Division of Dermatology and Venereology, Lund University, Lund SE-22184, Sweden; § Bioinformatics Institute (A*STAR), Singapore SG-138671; ¶ Department of Chemistry, University of Southampton, Southampton UK-SO17 1BJ, United Kingdom; ‖ Department of Pharmacy, University of Copenhagen, Copenhagen DK-2100, Denmark; ** Department of Biological Sciences, National University of Singapore, Singapore SG-117558; ‡‡ Department of Biomedical Sciences, Copenhagen Wound Healing Center, Bispebjerg Hospital, University of Copenhagen, Copenhagen DK-2400, Denmark

**Keywords:** antimicrobial peptide (AMP), innate immunity, aggregation, thrombin, lipopolysaccharide (LPS), peptides, bacteria, endotoxin, Toll-like receptor (TLR), inflammation, host defense, thrombin-derived C-terminal peptide (TCP)

## Abstract

Thrombin-derived C-terminal peptides (TCPs), including a major 11-kDa fragment (TCP96), are produced through cleavage by human neutrophil elastase and aggregate lipopolysaccharide (LPS) and the Gram-negative bacterium *Escherichia coli*. However, the physiological roles of TCP96 in controlling bacterial infections and reducing LPS-induced inflammation are unclear. Here, using various biophysical methods, *in silico* molecular modeling, microbiological and cellular assays, and animal models, we examined the structural features and functional roles of recombinant TCP96 (rTCP96) in the aggregation of multiple bacteria and the Toll-like receptor (TLR) agonists they produce. We found that rTCP96 aggregates both Gram-negative and Gram-positive bacteria, including *Staphylococcus aureus* and *Pseudomonas aeruginosa*, and their cell-wall components LPS, lipid A, and lipoteichoic acid (LTA). The Gram-negative bacteria *E. coli* and *P. aeruginosa* were particularly sensitive to aggregation-induced bacterial permeabilization and killing. As a proof of concept, we show that rTCP96 reduces LPS-induced NF-κB activation in human monocytes, as well as in mouse models of LPS-induced subcutaneous inflammation. Moreover, in a mouse model of subcutaneous inoculation with *P. aeruginosa*, rTCP96 reduced bacterial levels. Together, these results link TCP-mediated aggregation of endotoxins and bacteria *in vitro* to attenuation of inflammation and bacterial levels *in vivo*.

## Introduction

All wounds, whether caused by trauma, burns, or surgery, are at risk of becoming contaminated by bacteria, which could lead to infection. The ability to effectively counteract bacteria is of evolutionary significance to our survival. For this purpose, multiple host defense systems have evolved, such as multiple host defense peptides (1–4). Toll-like receptors (TLRs)^2^ have an important role in the innate immune system by detecting a broad range of pathogen-associated molecular patterns (5). For example, TLR4 is activated by lipopolysaccharides (LPS) from Gram-negative bacteria, and TLR2 is activated by lipoteichoic acid (LTA) and peptidoglycan (PGN) from Gram-positive bacteria (6). The sensing of microbes and their products by TLRs is crucial in early responses to infection. However, an excessive TLR activation is deleterious, causing localized inflammation, such as that found in infected wounds, but also severe systemic responses, as seen in sepsis (7). Therefore, clearance and control of not only bacteria but various TLR activators, such as LPS and LTA, is critical to maintaining an effective antibacterial response while maintaining control of inflammatory responses.

We have previously shown that proteolysis of thrombin by human neutrophil elastase (HNE) leads to the formation of thrombin-derived C-terminal peptides (TCPs) of roughly 2 kDa (8), which are present in wound fluids (9, 10) and have been demonstrated to exert antiendotoxic functions *in vitro* and *in vivo* (8, 10–14). Apart from these smaller fragments, proteolysis of thrombin by HNE also generates 11-kDa TCPs that are present in wounds. Recently, we have shown that such TCPs can aggregate both LPS and *Escherichia coli* bacteria, leading to killing of the bacteria, and subsequent phagocytosis in *in vitro* models (15). This study builds upon and extends our previous work with the aim of understanding of TCPs spectrum of interactions with TLR agonists and bacteria, and importantly, the physiological role *in vivo*.

Here, we demonstrate that a recombinant 96-amino acid TCP (rTCP96) aggregates both Gram-positive and Gram-negative bacteria and the products LPS, lipid A, and LTA. Finally, we show as a proof of principle, that such aggregation reduces LPS-induced inflammatory signaling as well as levels of *Pseudomonas aeruginosa* bacteria both *in vitro* as well as *in vivo* in experimental animal models.

## Results

### Antimicrobial effects of rTCP96

**Figure 1.**
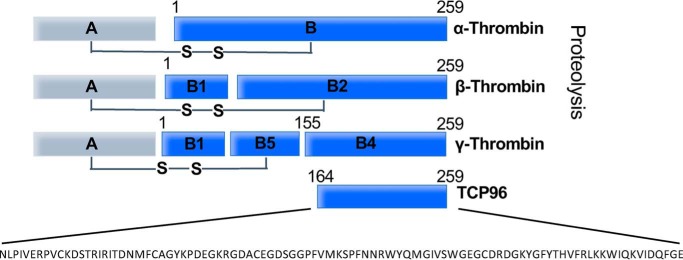
**Proteolysis of thrombin**. Illustration of proteolyzed thrombin products and the position and sequence of TCP96. The *numbers* indicate the amino acid position in the B chain.

**Figure 2.**
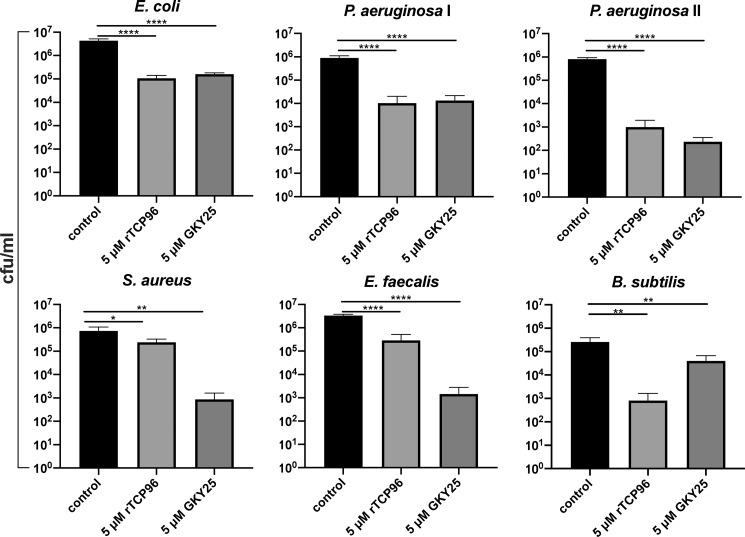
**Antimicrobial activity of rTCP96.** Viable count assay revealed a significant reduction in colony forming units of the indicated bacterial strains after treatment with 5 μm rTCP96. The strains used were *E. coli* ATCC 25922, *P. aeruginosa* ATCC 27853 (indicated by I and 9027 II), *S. aureus* ATCC 29213, *E. faecalis* ATCC 29212, and *B. subtilis* ATCC 6633. Results are expressed as the number of viable bacteria of 4 different experiments each carried out in triplicate. *, *p* ≤ 0.05; **, *p* ≤ 0.01; ****, *p* ≤ 0.0001. *p* values were determined relative untreated (control) bacteria using one-way ANOVA followed by Dunnett's multiple comparisons test.

**Figure 3.**
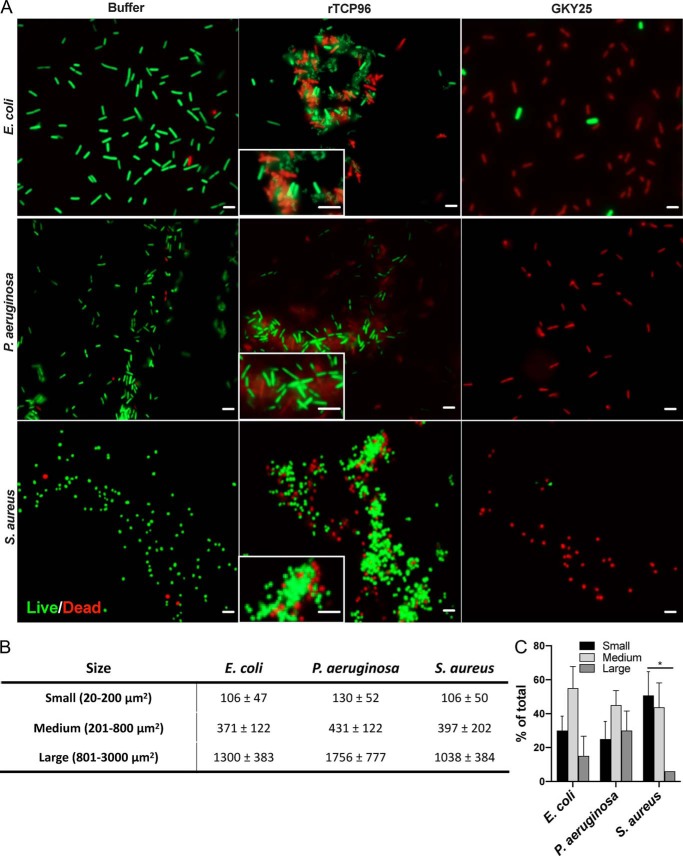
**Fluorescence microscopy analysis of bacterial viability.** *A,* visualization of live (*green*) and dead (*red*) bacteria. *E. coli* ATCC 25922, *P. aeruginosa* ATCC 27853, and *S. aureus* ATCC 29213 were subjected to 5 μm rTCP96 and bacterial viability in the aggregates was analyzed by LIVE/DEAD® BacLight^TM^ staining. The *insets* show a 6 times magnified region and the *scale bar* is 1 μm. The antimicrobial peptide GKY25 was used as positive control and did not aggregate bacteria. The *scale bar* is 2 μm. One representative image from three independent experiments is shown (*n* = 3). *B,* size distribution of aggregates for the indicated bacteria. *C,* the relative abundance of aggregates for the respective size class. For *B* and *C*, the analysis was performed by measuring the area of all the aggregates in 10 view fields from three different experiments. * indicate *p* ≤ 0.05 calculated using one-way ANOVA followed by Dunnett's multiple comparisons test.

TCP96 represents an HNE-generated fragment, which is nine amino acids shorter (from the N terminus) than the B4 chain of γ-thrombin (Fig. 1) (15). We recombinantly expressed TCP96 (rTCP96) and evaluated its antimicrobial effect on the Gram-positive *Staphylococcus aureus*, *Bacillus subtilis*, and *Enterococcus faecalis* and the Gram-negative *E. coli* and two *P. aeruginosa* isolates (indicated as I and II in Fig. 2). The results showed that rTCP96 reduced the levels of particularly the Gram-negative strains *E. coli* and *P. aeruginosa* by 100–1000–fold, whereas the reduction of the Gram-positive *S. aureus* and *E. faecalis* was, albeit statistically significant, less marked (Fig. 2). To analyze whether the killing was mediated by bacterial permeabilization, we next employed live/dead staining, which uses propidium iodide (red color) to detect loss of membrane integrity. As seen, rTCP96 aggregated the bacteria, and killed (red) bacterial cells were observed in the aggregates of *E. coli*, *P. aeruginosa*, and *S. aureus* (Fig. 3*A* and Fig. S1). The size distribution of the aggregates and the relative abundance of the respective size groups was recorded and is summarized in Fig. 3, *B* and *C*, respectively. The shorter TCP GKY25 killed the bacteria but did not cause aggregation (Fig. 3*A*, *rightmost panels*). We next selected *E. coli* and *S. aureus* bacteria for analysis using TEM after treatment with 5 μm rTCP96. Membrane breaks and perturbations, compatible with the results using the live/dead assay (Fig. 3*A* and Fig. S1) were observed (Fig. S2). Taken together, these results show that rTCP96 can induce aggregation and permeabilization of various Gram-negative and Gram-positive bacteria, leading to bacterial killing.

### Aggregation of rTCP96 in the presence of TLR ligands

**Figure 4.**
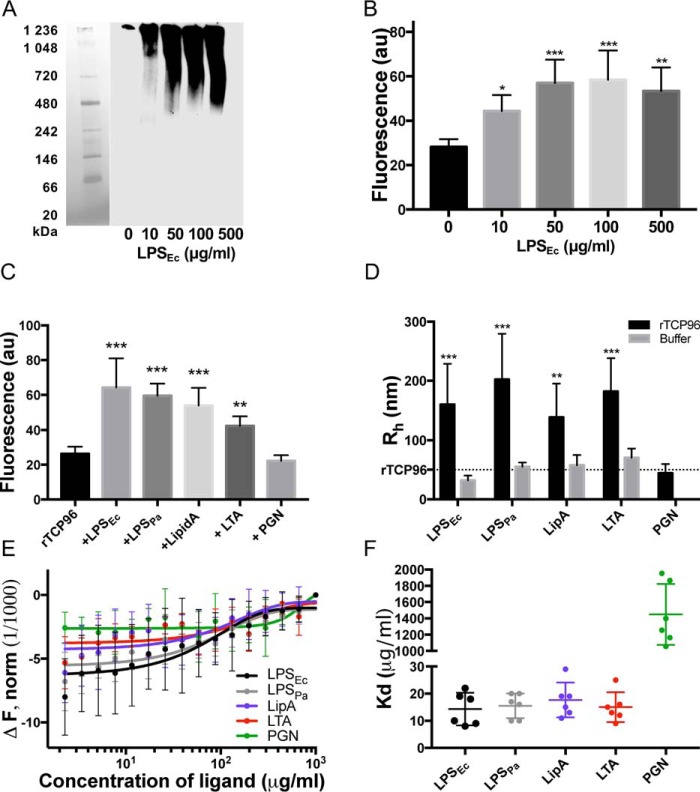
**Aggregation of rTCP96 in the presence of various agonists from Gram-positive and Gram-negative bacteria.** *A,* separation on Blue Native gels followed by Western blot analysis shows an increase of higher molecular complexes of rTCP96 (5 μm) with an increasing amount of LPS from *E. coli* (0–500 μg/ml). One representative image of four independent experiments is shown (*n* = 4). *B,* rTCP96 was incubated with LPS at the indicated concentrations. ThT assay demonstrates a significant increase of β-sheet structure in the studied concentration range for LPS (*n* = 6). *C,* ThT assay demonstrates aggregation of rTCP96 in the presence of LPS (from *E. coli* and *P. aeruginosa*), lipid A (from *E. coli*), LTA (from *S. aureus*) but not with PGN (from *S. aureus*) (*n* = 6). *D,* dynamic light scattering analysis of the samples analyzed in *C* is presented. For *B* and *C*, *, *p* ≤ 0.05; **, *p* ≤ 0.01; ****, *p* ≤ 0.0001. *p* values were determined using one-way ANOVA with Dunnett's multiple comparison test, and for *D* with two-way ANOVA with Sidak's multiple comparison test (*n* = 6). *E,* MST analysis for study of interaction of rTCP96 (1 μm) with LPS (*E. coli*), LPS (*P. aeruginosa*), lipid A (*E. coli*), LTA (*S. aureus*), and PGN (*S. aureus*) is shown. *F, K_d_* values based on the MST analysis. The respective values are *K_d_* (μg/ml) = 14 ± 6, 16 ± 5, 18 ± 7, 15 ± 6, and 1449 ± 375, for the respective ligands. Mean ± S.D. values of six measurements are shown.

Next, we investigated the interaction between rTCP96 and various TLR ligands. We used Blue Native gels to determine the complex formations between rTCP96 (5 μm) and LPS (*E. coli*, 0 to 500 μg/ml) (Fig. 4*A*). Under the conditions used, rTCP96 alone is not detected in the gel, whereas increased amounts of LPS caused a significant increase in complexes migrating as ∼700 kDa and larger (Fig. 4*A*). Furthermore, we measured a significant increase of β-sheet structure/aggregation by detecting thioflavin T1 (ThT) fluorescence in the samples of rTCP96 (5 μm), which were treated with LPS (*E. coli*) in the concentration range from 10 to 500 μg/ml in Tris buffer, pH 7.4 (Fig. 4*B*). Based on the results from the ThT experiment, for the next set of experiments, we chose to use 5 μm rTCP96 and 50 μg/ml of TLR ligands in Tris buffer, pH 7.4. We performed the ThT assay to determine the increase of β-sheet structure in rTCP96 treated with the TLR ligands LPS (*E. coli*), LPS (*P. aeruginosa*), lipid A (*E. coli*), LTA (*S. aureus*), and PGN (*S. aureus*). We detected a significant increase in the β-sheet secondary structure of rTCP96, as reflected by an increase in ThT fluorescence in rTCP96 subjected to LPS (from *E. coli* and *P. aeruginosa*), lipid A (*E. coli*), and LTA (*S. aureus*). We did not observe any changes after addition of PGN (*S. aureus*) (Fig. 4*C*). Dynamic light scattering analyses corresponded with the results from the ThT assay and the electrophoresis results using Blue Native gel. A significant increase of hydrodynamic radius, compatible with the observed interactions between rTCP96 and the TLR ligands, was detected in the presence of LPS (*E. coli*), LPS (*P. aeruginosa*), lipid A (*E. coli*), and LTA (*S. aureus*) but not in the presence of PGN (*S. aureus*) (Fig. 4*D*). Moreover, we employed microscale thermophoresis to measure the direct interaction between rTCP96 and TLR ligands in the solution (Fig. 4*E*). The *K_d_* (μg/ml) constants for LPS (*E. coli*), LPS (*P. aeruginosa*), lipid A (*E. coli*), LTA (*S. aureus*), and PGN (*S. aureus*) were 14 ± 6, 16 ± 5, 18 ± 7, 15 ± 6, and 1449 ± 375, respectively, demonstrating that rTCP96 exhibited significantly lower binding affinity to PGN when compared with the other TLR ligands (Fig. 4*F*).

### Structural changes in rTCP96 triggered by TLR ligands

**Figure 5.**
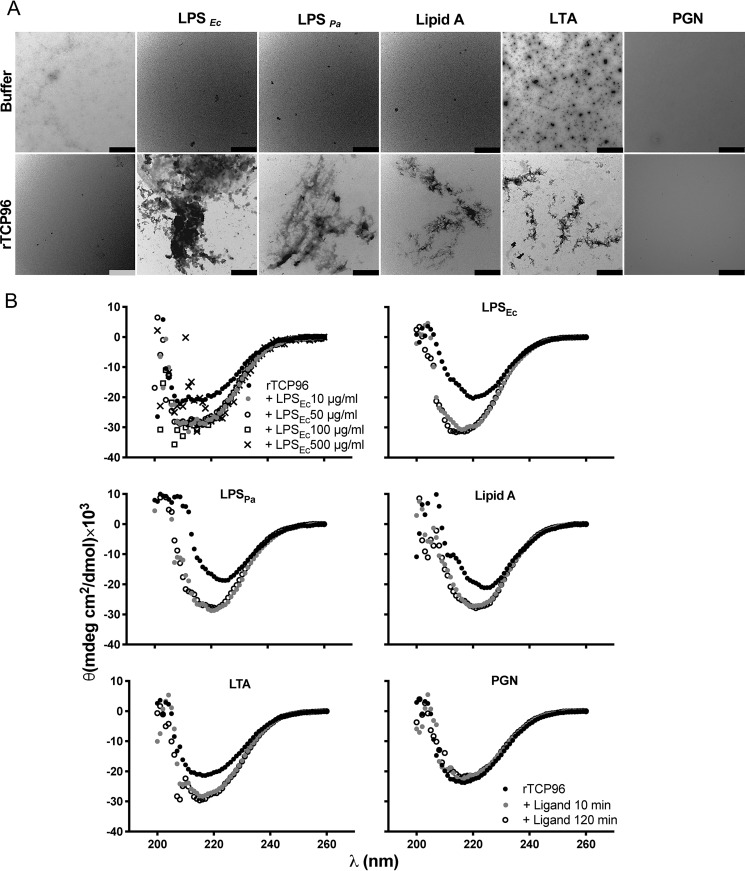
**Structural changes of rTCP96.** *A,* TEM-negative staining revealed amorphous aggregates of rTCP96 (size from 0.5 to 5 μm) after incubation with LPS from *E. coli* (Ec) and *P. aeruginosa* (*Pa*), lipid A (from *E. coli*), and LTA (from *S. aureus*). Amorphous aggregates of rTCP96 were not observed in the samples treated with PGN (*S. aureus*). One representative image from three independent experiments is shown (*n* = 3). The *scale bar* is 1 μm. *B,* CD was used to detect an increase of β-sheet structures in rTCP96 after incubation with LPS from *E. coli* (*Ec*) and *P. aeruginosa* (*Pa*), lipid A (from *E. coli*), and LTA (from *S. aureus*). PGN (from *S. aureus*) did not affect the secondary structure of rTCP96 (*n* = 3).

Next, we used TEM to visualize amorphous aggregates of rTCP96 (of sizes from 0.5 to 5 μm), which were formed after incubation with the different TLR ligands LPS, Lipid A, and LTA (Fig. 5*A*). rTCP96 did not aggregate in the presence of PGN. Correspondingly, an increase of β-sheet structure of rTCP96 was detected by CD measurements in the samples incubated with the two LPS forms, lipid A, and LTA (*S. aureus*). As above, no significant difference in the secondary structure of rTCP96 treated by PGN was observed (Fig. 5*B*). Inflammatory local environments can have a low pH (16, 17). We therefore next investigated aggregation and structural changes of rTCP96 at pH 5.5. Using the ThT assay we found that LPS, lipid A, and LTA also induced β-sheet formation at low pH (Fig. S3*A*). Correspondingly, CD analysis at pH 5.5 demonstrated changes in secondary structure of rTCP96 in the presence of the TLR ligands LPS, lipid A, and LTA (Fig. S3*B*).

### Molecular simulations of the TCP96-LPS and -LTA interaction

**Figure 6.**
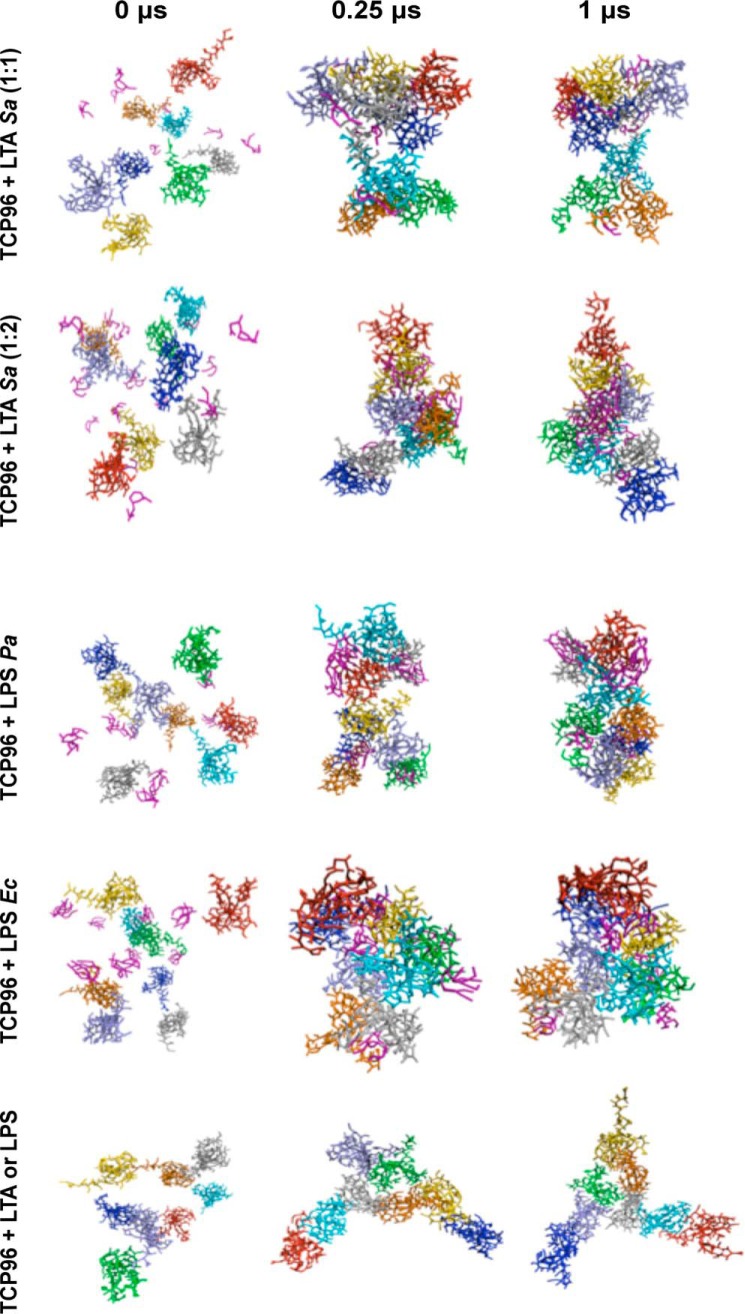
**Computational prediction of co-aggregation of TCP96 fragments from random starting locations, in presence/absence of different microbial products.** Snapshots show LTA/LPS lipids (*purple*) with surrounding TCP96 molecules in multiple colors, at the start, at 0.25 μs (when most of the molecules had undergone aggregation), and at the end of each simulation. *Ec*, *E. coli*; *Pa*, *P. aeruginosa*; *Sa*, *S. aureus.*

**Figure 7.**
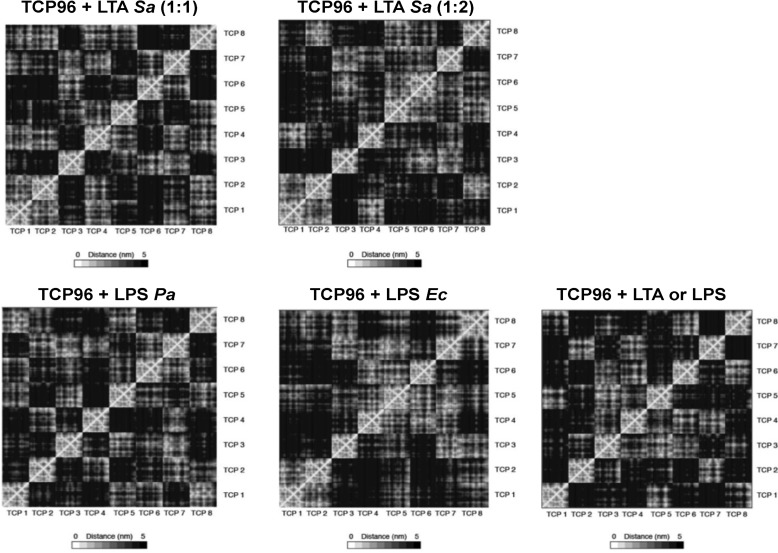
**Mean intermolecular distances between TCP96 fragments following co-aggregation simulations in the presence/absence of various microbial products.** The panels indicate pairwise intermolecular distances between all eight TCP96 molecules in the simulation box, ranging from close contact (*white*) to more separated (*black*). *Bright regions* of the panels signify larger contact areas between molecules. *Dark areas* indicate that the respective molecules do not interact. Increased aggregation is apparent through the appearance of additional bright areas in the system containing LTA/LPS. Note that the bright area through the center of each matrix is due to self-contact. *Ec*, *E. coli*; *Pa*, *P. aeruginosa*; *Sa*, *S. aureus.*

CG simulations enabled us to study the large-scale aggregation propensity for TCP96 fragments in the presence of the lipid core components from different microbial products, including LPS from *P. aeruginosa* and *E. coli*, and LTA from *S. aureus*. The simulations in the presence of all molecules displayed an increase in aggregation over time, driven primarily by hydrophobic interactions (Fig. 6). Pairwise distance analysis supported visual analysis (Fig. 7), with LPS derived from different species forming stable co-aggregates in a 1:1 ratio. Notably, the 1:2 ratio of TCP96 with LTA lipids exhibited a greater propensity for aggregation compared with the 1:1 ratio, consistent with the smaller size of the lipidic component of LTA compared with that of LPS.

The RDF for TCP96-TCP96 interactions was next calculated, as a measure of the mean variation in density as a function of distance from the protein fragments (Fig. S4). The RDFs indicate that TCP96s come into closer contact in the presence of LTA or LPS compared with when they are incubated alone. Based on the first peak in the RDF profiles, these differences in contact distances were shown to be significantly different, and also confirm that for each simulation the co-aggregation process had converged following the first 0.2 μs of simulation (Table S1). Furthermore, the data lend additional support to the observation that a higher concentration of LTA is required compared with LPS for efficient TCP96 aggregation.

### Effects of rTCP96 on endotoxin response in monocytes

**Figure 8.**
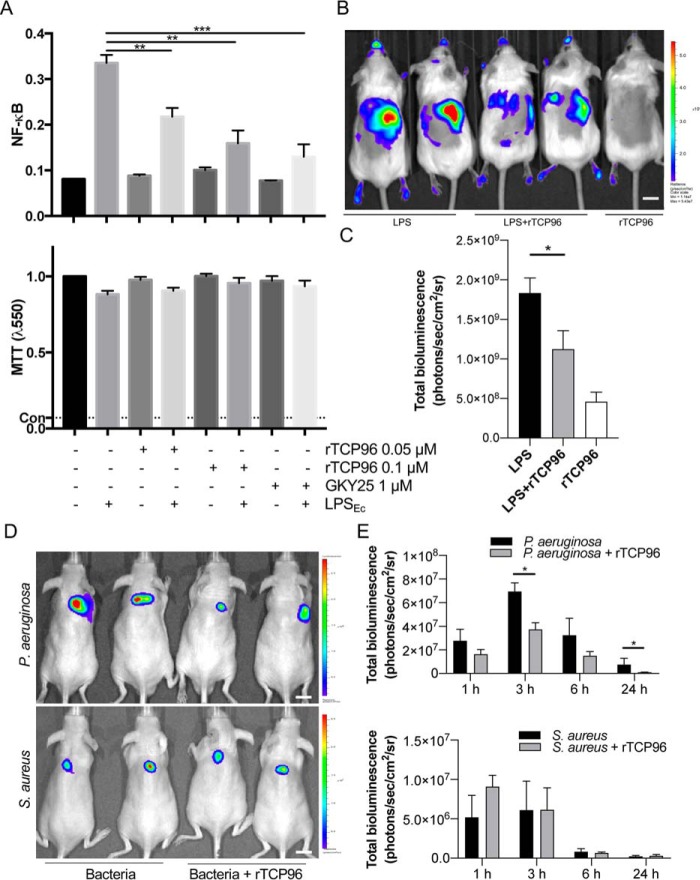
**Antiendotoxic effect of rTCP96 *in vitro* and *in vivo*.** *A,* THP-1-XBlue-CD14 cells were treated with rTCP96, LPS from *E. coli* (*Ec*), or a combination of both. rTCP96 yielded a significant reduction of activation of NF-κB/AP-1 (14). **, *p* ≤ 0.01; ****, *p* ≤ 0.0001. MTT viability assay for analysis of toxic effects of rTCP96 on THP-1 cells is shown. The *dotted line* (*Con*) represents positive control of dead cells. The mean ± S.D. values of five measurements are shown. *p* values were determined using one-way ANOVA with Dunnett's multiple comparison test. *B,* NF-κB activation in the NF-κB-RE-luc random transgenic mouse model was analyzed by the IVIS imaging system. LPS, or LPS aggregated with rTCP96 (*LPS* + *rTCP96*), were injected subcutaneously and the NF-κB response was imaged after an incubation period of 3 h. The *scale bar* is 1 cm. *C,* total radiance from of the experiment illustrated in *B* was measured. A significant reduction of NF-κB activation was observed in mice treated with rTCP96-LPS compared with LPS treatment alone. The mean ± S.D. values of five to seven measurements are shown. *, *p* ≤ 0.05. One-way ANOVA with Dunnett's multiple comparison test was used. *D,* effects of rTCP96 in a mouse model of subcutaneous infection are illustrated. *In vivo* infection imaging in a mouse model of subcutaneous infection. Bioluminescent *P. aeruginosa* or *S. aureus* bacteria (3 × 10^8^ cfu/ml) was incubated with rTCP96 or buffer only and deposited subcutaneously in the dorsum of SKH1 mice. Bioluminescence intensity was noninvasively analyzed using the IVIS bioimaging system. Representative images show bacterial luminescence at 3 h post-infection. The *scale bar* is 1 cm. *E,* the bar chart shows measured bioluminescence intensity emitted by the bacteria at 1, 3, 6, and 24 h post-infection. All *in vivo* data are presented as the mean ± S.E. (*n* = 5–7 mice). *, *p* ≤ 0.05. *p* values were determined using the Mann-Whitney *U* test.

We next used reporter THP-1 monocytes to detect effects on LPS signaling by rTCP96. rTCP96 significantly reduced the activation of NF-κB/AP-1 triggered by *E. coli* LPS (Fig. 8*A*). The MTT viability assay did not show any significant cytotoxic effect of rTCP96 on THP-1 cells, which suggests that the reduction in the NF-κB/AP-1 activation was due to the neutralizing effect of rTCP96 on LPS and not by any rTCP96-mediated toxic effects (Fig. 8*A*).

### Effects of rTCP96 on endotoxin and bacteria in vivo

We next explored whether rTCP96 could suppress LPS-triggered local inflammation *in vivo*. For this, we utilized the (NF-κB-RE-Luc)-Xen reporter mouse model and studied the effects of rTCP96 on subcutaneous inflammation induced by LPS. (NF-κB-RE-Luc)-Xen reporter mice carry a transgene containing six NF-κB-responsive elements and a modified firefly luciferase cDNA. The reporter gene is inducible by LPS and helps in *in vivo* studies of transcriptional regulation of the NF-κB gene. LPS (25 μg) was subcutaneously injected into the mice, either alone or with rTCP96 (25 μg). The luminescent signal after subsequent addition of luciferin, reporting NF-κB activation, was recorded using live bioimaging (IVIS Spectrum) (Fig. 8*B*). We detected a significant reduction of NF-κB activation after 3 h in mice co-treated with rTCP96-LPS when compared with LPS treatment alone. rTCP96 alone did not yield any significant increase in NF-κB activation (Fig. 8*C*). In the next experimental model, we wanted to simulate a situation of direct contamination with bacteria. Bioluminescent *P. aeruginosa* or *S. aureus* bacteria (10^6^ cfu, cfu/animal) were incubated with buffer or rTCP96 and immediately injected subcutaneously in SKH1 mice. In this model, the bacterial dose used causes a transient, and self-limiting bacterial infection. The results showed that rTCP96 reduced the bacterial load of *P. aeruginosa*, as assessed by *in vivo* bioimaging (Fig. 8, *D* and *E*). However, no significant reduction of *S. aureus* was observed (Fig. 8, *D* and *E*).

## Discussion

In this study, we present evidence that rTCP96 aggregates not only Gram-negative *E. coli* but also other Gram-negative bacteria, such as *P. aeruginosa*, as well as Gram-positive bacteria, such as *S. aureus*. Although both Gram-negative and Gram-positive bacteria were aggregated *in vitro*, we observed that bacterial killing was most pronounced for the Gram-negative *E. coli* and *P. aeruginosa in vitro*. Correspondingly, rTCP96 only reduced *P. aeruginosa* in an experimental model of subcutaneous inoculation of *P. aeruginosa*, whereas no effects on *S. aureus* were observed in the *in vivo* model. From a clinical perspective, this observation corresponds with the well-established fact that *E. coli* and *P. aeruginosa* are less frequent as infective agents in acute surgical wounds. Notably, the majority of surgical site infections are caused by Gram-positive bacteria, of which one major agent is *S. aureus* (18, 19). Although speculative, these observations suggest that TCP-mediated aggregation at physiological conditions, such as in wounds, may preferentially target bacteria, such as *E. coli* and *P. aeruginosa.*

We also show that TLR4 agonists such as LPS and lipid A from various bacteria (*E. coli* and *P. aeruginosa*) cause aggregation of rTCP96. Notably, the TLR2 agonist LTA from *S. aureus* exhibited a similar aggregating effect on rTCP96. On the other hand, PGN (from *S. aureus*), which is a TLR2 agonist, did not exert any aggregating effects on rTCP96. In this context, it is notable that a major chemical difference between PGN and the other ligands is the lack of an acyl tail component in PGN. Hence, the data suggest that the hydrophobic lipid tails of lipid A, LPS, and LTA seem to be crucial in mediating the aggregation with TCP96, and likewise in binding to TLR hydrophobic pockets. Of particular importance was that we observed an anti-inflammatory effect of rTCP96, as it significantly reduced LPS-induced TLR4 activation of human monocytes *in vitro*, indicating that confinement of LPS leads to reduced inflammatory signaling. Importantly, this *in vitro* observation was translated to the *in vivo* situation, showing that TCP96 can also reduce NF-κB activation in the experimental model of endotoxin-induced inflammation in NF-κB-RE-luc mice.

Taken together, the present work on aggregation of TCPs induced by bacteria and their products, therefore, adds a further role to proteolyzed thrombin fragments in aggregation and amyloid formation for rapid confinement of endotoxins and microbes. This connection between host defense and aggregation suggest that control of bacteria and its products is a common theme for many amyloidogenic proteins (20), such as β-amyloid-peptide variants (21, 22), peptides from β-amyloid precursor protein, and the prion protein (13, 23). Moreover, eosinophilic cationic protein, known to be released from eosinophils, aggregates and kills bacteria *in vitro* (24). It remains to be investigated whether these other proteins and peptides mediate similar activities as reported here for TCP96.

**Figure 9.**
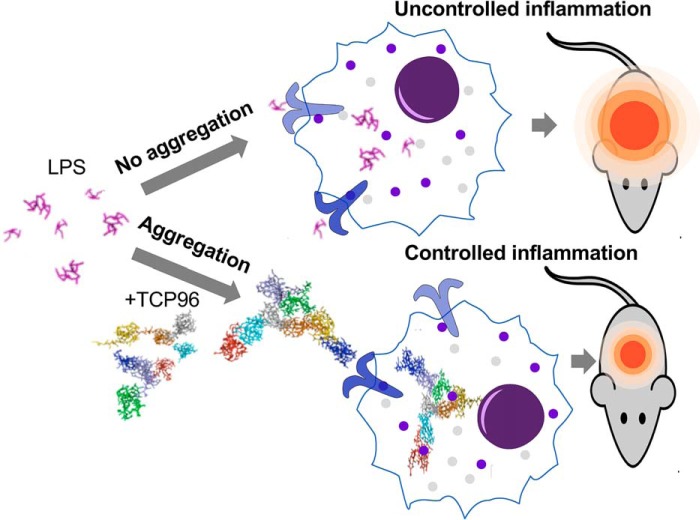
**Summary of TCP96 function.** Aggregation and confinement of LPS leads to attenuated inflammation *in vivo*.

Worldwide antimicrobial resistance is an increasingly serious threat to global public health that requires the urgent discovery of new therapeutic approaches (25–27). The activities of TCP96 on LPS delineate an endogenous mechanism by which aggregation-prone TCPs facilitate and control inflammation. From an evolutionary perspective, this activity is both logical and beneficial from an organism's point of view, illustrating that it is better to localize and attenuate inflammation than not to contain it (Fig. 9). This observation is of interest, as this suggests that potentially lethal systemic reactions, such as seen in endotoxin-induced shock, can potentially be avoided. Future *in vivo* work utilizing systemic models of endotoxin shock and bacterial infections with both Gram-negative as Gram-positive pathogens are mandated to explore the therapeutic potential of anti-infective concepts based on aggregation induced confinement.

## Experimental procedures

### Peptide

The thrombin-derived peptide GKY25 (GKYGFYTHVFRLKKWIQKVIDQFGE) was synthesized by Biopeptide Co., Inc. We confirmed the purity (over 95%) via mass spectral analysis (MALDI-TOF Voyager).

### Microorganisms

*E. coli* ATCC 25922, *S. aureus* ATCC 29213, *S. aureus* 229, *P. aeruginosa* ATCC 9027, *P. aeruginosa* ATCC 27853, *P. aeruginosa XEN 41*, *B. subtilis* ATCC 6633, and *E. faecalis* ATCC 29212 bacterial strains were purchased from LGC (UK).

### Cell culture

THP-1 cells (ATCC) were cultured in Roswell Park Memorial Institute 1640 Medium-GlutaMAX-1 (Gibco, Life Technology Ltd., UK), the medium was supplemented with 10% (v/v) heat-inactivated FBS (Invitrogen) and 1% (v/v) antibiotic-antimycotic solution (Invitrogen) at 37 °C in 5% CO_2_.

### TLR ligands

LPS from *E. coli*, LPS from *P. aeruginosa*, and lipid A from *E. coli* were purchased from Sigma-Aldrich. LTA and PGN from *S. aureus* were purchased from InvivoGen.

### Animals

BALB/c tg(NF-κB-RE-Luc)-Xen reporter male mice and SKH-1 hairless male mice, purchased from Taconic Biosciences, were used for all experiments. The animals were housed under standard conditions of light and temperature and had free access to standard laboratory chow and water.

### Purification of rTCP96

A bacterial expression system consisting of pET-15b plasmid in *E. coli* strain BL21 codon plus (DE3) *RIPL* (Invitrogen) was used to produce the rTCP96. We cultivated the bacteria in LB broth (Sigma-Aldrich) supplemented with 34 μg/ml of chloramphenicol and 100 μg/ml of carbomycin. Isopropyl 1-thio-β-d-galactopyranoside (400 μm; VWR), added at the mid-log phase, was used to induce peptide production in the bacterial system. The rTCP96 peptides were extracted and purified by immobilized metal affinity chromatography (nickel-nitrilotriacetic acid-agarose, Invitrogen) under denaturing conditions (8 m urea, 10 mm Tris, pH 7.4), extensively washed with 20 mm imidazole in 8 m urea, 10 mm Tris, pH 7.4, and then eluted by stepwise increasing concentrations of imidazole (200 mm). rTCP96 was desalted in 10 mm Tris, pH 7.4, by stepwise dialysis and concentrated using a 3-kDa molecular mass cut-off Amicon ultracentrifugal filter device (Millipore, Germany) and stored at 4 °C prior to use. Peptide purity was confirmed via Tricine gel electrophoresis followed by Gel Code Blue Safe Protein staining (Thermo Scientific) and Western blotting. The protein concentration was determined by Nanodrop (ND 1000, Thermo Scientific) (15).

### Native gel analysis (BN-PAGE)

We detected the interactions of rTCP96 and TLR ligands by BN-PAGE and Western blot analysis. rTCP96 (5 μm) was incubated with TLR ligands for 30 min at 37 °C. The samples were loaded under reducing conditions on BN-PAGE (Native PAGE BisTris Gels System 4–16%, Invitrogen) according to the manufacturer's instructions, which we followed by Western blotting. Although the complex formation is known to be a process involving several intermediate steps, this assay is accepted in the field as an approximate measure of the binding capability between peptides and ligands.

### Viable count assay

To determine the antibacterial activity of rTCP96, we used *E. coli* ATCC 25922, *S. aureus* ATCC 29213, *P. aeruginosa* ATCC 27853 (indicated as Pa I), *P. aeruginosa* ATCC 9027 (indicated as Pa II), *B. subtilis* ATCC 6633, and *E. faecalis* ATCC 29212. The bacteria were grown to mid-logarithmic phase in 5 ml of Todd-Hewitt (TH) medium. The bacteria were centrifuged, washed, and suspended in 5 ml of 10 mm Tris buffer, pH 7.4. Next, the bacteria (10 μl, 3 × 10^8^ cfu; cfu/ml) were incubated with 5 μm rTCP96, 5 μm GKY25 (used as a positive control), or buffer control (10 mm Tris buffer, pH 7.4) for 2 h at 37 °C. A dilution series of the incubated samples were plated on TH agar plates, incubated overnight at 37 °C, and the cfu was calculated (15).

### Thioflavin T dye-binding assays

Amyloid formation was determined using the dye ThT. Thioflavin T preferentially binds to the β-sheet structures of amyloidogenic proteins/peptides. For examination of the concentration dependence of the aggregation, we incubated rTCP96 (5 μm) and LPS from *E. coli* (0, 10, 50, and 100 μg/ml) in buffer (10 mm Tris, pH 7.4, and 10 mm MES, pH 5.5) for 30 min at 37 °C. Moreover, rTCP96 was incubated for with 50 μg/ml of each ligand (LPS (*E. coli*), LPS (*P. aeruginosa*), lipid A (*E. coli*), LTA (*S. aureus*), and PGN (*S. aureus*)) for 30 min at 37 °C before measurements. Two hundred microliters of the materials were incubated with 100 μm ThT for 15 min in the dark (ThT stock was 1 mm stored in the dark at 4 °C). We measured ThT fluorescence using a VICTOR3 Multilabel Plate Counter spectrofluorometer (PerkinElmer Life Sciences) at an excitation of 450 nm, with excitation and emission slit widths of 10 nm. The baseline (10 mm Tris, pH 7.4, or 10 mm MES, pH 5.5, buffer, LPS (*E. coli*), LPS (*P. aeruginosa*), lipid A (*E. coli*), LTA (*S. aureus*), and PGN (*S. aureus*)) was subtracted from the signal of each sample (15, 28).

### Circular dichroism spectroscopy

We performed circular dichroism (CD) measurements on a Jasco J-810 spectropolarimeter (Jasco) equipped with a Jasco CDF-426S Peltier set to 25 °C. The peptides were diluted to 5 μm in buffer (Tris, 10 mm, pH 7.4, and MES, pH 5.5) and incubated with 10–300 μg/ml of LPS for 30 min, placed in a 10-mm quartz cuvette and, after extensive purging with nitrogen, scanned over the wavelength interval at 200–260 nm (scan speed: 20 nm/min). We calculated the averages of five scans for each sample. For examination of time dependence, rTCP96 (5 μm) was incubated for 10 and 120 min at 37 °C in the absence or presence of TLR ligands (50 μg/ml) in 10 mm Tris, pH 7.4. The baseline (10 mm Tris, pH 7.4, or 10 mm MES, pH 5.5, buffer, LPS (*E. coli*), LPS (*P. aeruginosa*), lipid A (*E. coli*), LTA (*S. aureus*), and PGN (*S. aureus*)) was subtracted from the spectra of each sample (15, 29).

### Transmission electron microscopy

We visualized the aggregates formed by rTCP96 (5 μm) in the presence of ligands (50 μg/ml), such as LPS (*E. coli*), LPS (*P. aeruginosa*), lipid A (*E. coli*), LTA (*S. aureus*), and PGN (*S. aureus*) during incubation for 30 min at 37 °C. We examined 10 view fields (magnification ×4200) of the mounted samples on the grid (pitch 62 μm) from three independent sample preparations using TEM (Jeol Jem 1230; Jeol, Japan) in combination with negative staining. The samples were adsorbed onto carbon-coated grids (copper mesh, 400) for 60 s and stained with 7 μl of 2% uranyl acetate for 20 s. The grids were rendered hydrophilic via glow discharge at low air pressure (15).

### Fluorescence microscopy analysis of bacterial aggregates

The viability of *E. coli* ATCC 25922, *S. aureus* ATCC 29213, and *P. aeruginosa* ATCC 27853 in the aggregates was assessed by using the LIVE/DEAD® BacLight^TM^ Bacterial Viability kit (Invitrogen, Molecular Probes, Carlsbad, CA). For this purpose, the bacterial suspension was prepared as described above for VCA. Bacteria were then treated by 5 μm rTCP96 or 5 μm GKY25 in 10 mm Tris, pH 7.4. The buffer was used for control. After 2 h, the samples were mixed with 3 μl of the dye mixture for each ml of the bacterial suspension, as reported on the manufacturer's protocol, and incubated in the dark at room temperature for 15 min. At the end of incubation, 5 μl of the stained bacterial suspension were trapped between a slide and an 18-mm square coverslip.

We examined 10 view fields (1 × 1 mm) of the mounted samples from three independent sample preparations using a Zeiss AxioScope A.1 fluorescence microscope (objectives: Zeiss EC Plan-Neofluar ×100/1.3 oil; camera: Zeiss AxioCam MRm; acquisition software: Zeiss Zen 2.6 (blue edition). For the analysis we measured the area of all aggregates possible to distinguish in each picture (43 for *E. coli*, 42 for *P. aeruginosa,* and 67 for *S. aureus*).

### Microscale thermophoresis

We performed MST analysis using a NanoTemper Monolith NT.115 apparatus (Nano Temper Technologies, Germany). We used a Monolith NT Protein labeling kit RED-NHS (Nano Temper Technologies) to label 5 μm rTCP96 according to the manufacturer's protocol. A constant amount of 2 μm rTCP96 was mixed with increasing concentrations of LPS (*E. coli*), LPS (*P. aeruginosa*), lipid A (*E. coli*), LTA (*S. aureus*), and PGN (*S. aureus*) in Tris buffer (10 mm, pH 7.4). Next, 10 μl of each sample was loaded into standard glass capillaries (Monolith NT Capillaries, Nano Temper Technologies), and we performed the MST analysis (settings for the light-emitting diode and IR laser were 80%). *K_d_* constants were analyzed using MST software MO Affinity Analysis version 2.2.4 (15).

### Dynamic light scattering

We performed dynamic light scattering (DLS) measurements to determine the hydrodynamic radii of rTCP96 with or without ligands: LPS (*E. coli*), LPS (*P. aeruginosa*), lipid A (*E. coli*), LTA (*S. aureus*), and PGN (*S. aureus*). Wyatt QELS (Quasi-Elastic Light Scattering, Wyatt Technology Corporation) and Dawn EOS (enhance optical system, Wyatt Technology Corp.) equipped with a temperature-controlled microsampler instrument were used for DLS measurements. We incubated the samples for 30 min at 37 °C under reducing conditions, and the scattered light was detected at 18 different angles simultaneously. Before the experiment, all samples were filtered through 0.22 μm pore-sized microfilters (Sartorius, Germany). Aliquots of samples were manually loaded into the flow cell and measured at 37 °C. All samples (1 μm rTCP96 with or without ligands: LPS (*E. coli*), LPS (*P. aeruginosa*), lipid A (*E. coli*), LTA (*S. aureus*), and PGN (*S. aureus*)) were measured at least 15 times. GKY25 peptide was used as a negative control and analyzed under the same conditions. For evaluation of time dependence of the aggregation, rTCP96 (1 μm) was incubated for 10, 30, 60, and 120 min at 37 °C in the absence or presence of LPS (*E. coli*), LPS (*P. aeruginosa*), lipid A (*E. coli*), LTA (*S. aureus*), and PGN (*S. aureus*) (5 μg/ml) in 10 mm Tris, pH 7.4. The hydrodynamic radii were analyzed by Astra V software using Zimm modeling (15).

### Molecular dynamics simulations

The coarse-grained (CG) models of TCP96 and *E. coli* LPS were taken from previously published work, with the closely related *P. aeruginosa* LPS model derived from that reported in Ref. 15. This involved switching the GL0 and GL5 beads in our original *E. coli* LPS CG model and shortening the lengths of the carbon lipid tails (30). The initial CG model for *S. aureus* LTA was initially constructed based on an atomic model kindly provided by Dr. T. J. Piggot. This model did not include the extended glycerol-phosphate units but was restricted to the hydrophobic diacylglycerol component and carbon lipid tails, which anchor LTA to the cell membrane, and are likely to be key in the aggregation of the molecule, as well as interacting with the hydrophobic TLR2 ligand-binding pocket (31). To study the aggregation behavior of TCP with different lipids, 5 × 1-μs simulation replicas were run with eight TCP96 fragments in the presence of: (i) eight *S. aureus* LTA molecules; (ii) eight *P. aeruginosa* LPS molecules; or (iii) eight *E. coli* LPS molecules. Also, 5 × 1-μs simulations of eight TCP96 fragments with 16 *S. aureus* LTA molecules were run, to check for possible side effects upon aggregation, given that the lipid component of LTA is significantly smaller than that of LPS. In all systems, the proteins and microbial products were randomly placed and solvated with water and neutralizing Na^+^ and Cl^−^ ions. The simulations were run using the MARTINI force field (32) at 313 K and 1 bar, which was kept constant using the Berensden algorithm (33). All simulations and analysis were carried out using Gromacs 2018 (34), whereas rendering was performed using VMD (35). The pairwise distances between all the residues within the TCP molecules were determined and averaged. The radial distribution function (RDF) for TCP-TCP interactions was calculated for each simulation. For all analysis, each simulation was divided into blocks of 0.2 μs, discarding the first block to account for equilibration.

### NF-κB activity assay

NF-κB/AP-1 activation, in THP-1-XBlue-CD14 reporter monocytes, was determined after 20–24 h of incubation according to the manufacturer's protocol (InvivoGen). Briefly, 1 × 10^6^ cells/ml in RPMI were seeded in 96-well-plates (180 μl) and incubated with peptides (GKY25 1 μm; rTCP96 0.5–0.05 μm), LPS (10 ng/ml), or both overnight at 37 °C, 5% CO_2_ in a total volume of 200 μl. The following day, the activation of NF-κB/AP-1 was analyzed as the secretion of embryonic alkaline phosphatase. The supernatant (20 μl) from the cells was transferred to 96-well-plates, and 180 μl of Quanti-Blue was added. The plates were incubated for 2 h at 37 °C, and the absorbance was measured at 600 nm in a VICTOR3 Multilabel Plate Counter spectrofluorometer.

### MTT viability assay

Sterile-filtered MTT (Sigma-Aldrich) solution (5 mg/ml in PBS) was stored in dark at −20 °C until usage. We added 20 μl of MTT solution to the remaining overnight culture of THP-1-XBlue-CD14 reporter monocytes from the above NF-κB activity assay in 96-well-plates, which were incubated at 37 °C. The supernatant was then removed, and the blue formazan product generated in cells was dissolved by the addition of 100 μl of 100% DMSO in each well. The plates were then gently shaken for 10 min at room temperature to dissolve the precipitates. The absorbance was measured at 550 nm in a VICTOR3 Multilabel Plate Counter spectrofluorometer.

### Mouse inflammation model

BALB/c tg(NF-κB-RE-Luc)-Xen reporter mice (Taconic, 10–12 weeks old) were used to study the immunomodulatory effects of rTCP96 (25 μg) after subcutaneous co-treatment with LPS (*E. coli*, 25 μg). The samples were preincubated for 30 min at 37 °C before injection. The dorsums of the mice (5 to 6 mice per treatment group) were carefully shaved and cleaned. Mice were anesthetized with isoflurane, and 200 μl of the sample was injected subcutaneously. The animals were immediately transferred to individually ventilated cages and imaged 3 h later. We used an In Vivo Imaging System (IVIS Spectrum, PerkinElmer Life Sciences) for determination of NF-κB activation, which plays a key role in the regulation of immune response during infection. Bioluminescence from the mice was detected and quantified using Living Image 4.0 Software (PerkinElmer Life Sciences). Fifteen minutes before the IVIS imaging, mice were intraperitoneally given 100 μl of d-luciferin (150 mg/kg body weight) (14).

### Mouse model of subcutaneous infection

Male SKH-1 hairless mice (12 weeks old), were anesthetized using a mixture of 2% isoflurane and oxygen. Overnight cultures of bioluminescent bacteria, *P. aeruginosa* Xen41 or *S. aureus* 229, were refreshed and grown to mid-logarithmic phase in TH media. Bacteria were washed for 15 min (5.6 × 1000 rpm) and diluted with 10 mm Tris buffer, pH 7.4. rTCP96 (5 μm) was then mixed with 10^6^ cfu of the bacteria and incubated for 2 h at 37 °C. A total of 100 μl of mixture was injected subcutaneously into the mouse dorsum. *In vivo* bacterial infection was imaged by measuring bioluminescence in anesthetized mice using IVIS imaging and the data obtained were analyzed using Living Image 4.0 Software (PerkinElmer Life Sciences). 5 to 6 mice per treatment group were used (14).

### Ethics statement

All animal experiments were performed according to the Swedish Animal Welfare Act SFS 1988:534 and were approved by the Animal Ethics Committee of Malmö/Lund, Sweden (permit numbers M88-91/14, M5934-19, and M8871-19). Animals were kept under standard conditions of light and temperature and water *ad libitum*.

### Statistical analysis

The graphs of VCA, ThT, and DLS are presented as mean ± S.D. from at least three independent experiments. We assessed differences in these assays using one-way ANOVA with Dunnett's multiple comparison tests and two-way ANOVA with Sidak's multiple comparison tests. All of the data were analyzed using GraphPad Prism 8 (GraphPad Software, Inc.). The *p* values less than 0.05 were considered to be statistically significant (*, *p* < 0.05; **, *p* < 0.01; ***, *p* < 0.001; and ****, *p* < 0.0001).

## Author contributions

J. P. and A. S. conceptualization; J. P. and R. G. H. data curation; J. P., G. P., R. G. H., E. W. M., and M. P. formal analysis; J. P., R. G. H., P. J. B., and A. S. supervision; J. P., P. J. B., and A. S. funding acquisition; J. P., G. P., P. J. B., and M. P. validation; J. P., G. P., M. J. v. d. P., P. J. B., M. P., and A. S. investigation; J. P., G. P., R. G. H., and E. W. M. visualization; J. P., G. P., and M. P. methodology; J. P. and A. S. writing-original draft; J. P., G. P., R. G. H., M. J. v. d. P., P. J. B., M. P., and A. S. writing-review and editing; A. S. resources.

## Supplementary Material

Supporting Information

## Footnotes

2 The abbreviations used are:

TLR: Toll-like receptor
LPS: lipopolysaccharide
PGN: peptidoglycan
LTA: lipoteichoic acid
HNE: human neutrophil elastase
TCP: thrombin-derived C-terminal peptide
TEM: transmission electron microscope
ThT: thioflavin T
MTT: 3-(4,5-dimethylthiazol-2-yl)-2,5-diphenyltetrazolium bromide
cfu: colony-forming unit
Tricine: *N*-[2-hydroxy-1,1-bis(hydroxymethyl)ethyl]glycine
BN-PAGE: Blue Native-PAGE
BisTris: 2-[bis(2-hydroxyethyl)amino]-2-(hydroxymethyl)propane-1,3-diol
DLS: dynamic light scattering
CG: coarse grained
RDF: radial distribution function
MST: microscale thermophoresis
ANOVA: analysis of variance.
