# Supplementary material for: Thrombin-derived C-terminal fragments aggregate and scavenge bacteria and their proinflammatory products
Source: J Biol Chem. 2020 Feb 7;295(11):3417–30. doi: 10.1074/jbc.RA120.012741 (PMC7076200; doi:10.1074/jbc.RA120.012741)
Supplement: Supporting Information [file supp_295_11_3417__index.html]

Thrombin-derived C-terminal fragments aggregate and scavenge bacteria and their proinflammatory products — Thrombin fragments aggregate bacteria — Thrombin-derived C-terminal fragments aggregate and scavenge bacteria and their proinflammatory products — Thrombin fragments aggregate bacteria — Supporting Information 

# Thrombin-derived C-terminal fragments aggregate and scavenge bacteria and their proinflammatory products

## Supporting Information

- Supporting Information (to be published online) - Figures and table
