# Supplementary material for: Thrombin-derived C-terminal fragments aggregate and scavenge bacteria and their proinflammatory products
Source: J Biol Chem. 2020 Feb 7;295(11):3417–30. doi: 10.1074/jbc.RA120.012741 (PMC7076200; doi:10.1074/jbc.RA120.012741)
Supplement: Supporting Information [file supp_RA120.012741_158362_1_supp_471205_q566q2.pdf]

Supplementary Information.

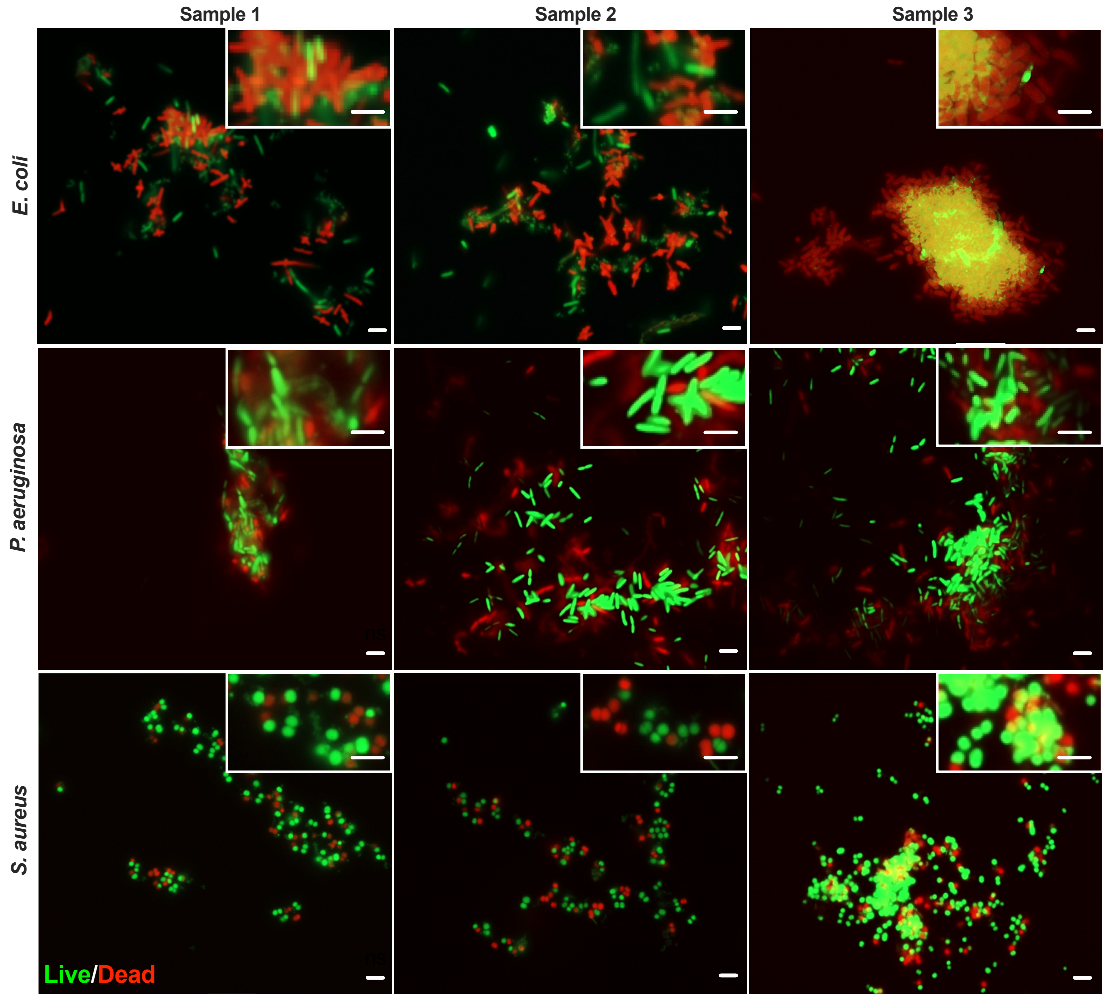

Figure S1. Fluorescence microscopy analysis of bacterial viability. Representative images of *Escherichia coli* ATCC 25922, *Pseudomonas aeruginosa* ATCC 27853 and *Staphylococcus aureus* ATCC 29213 subjected to rTCP96 (5 µM) followed by staining of the aggregates by LIVE/DEAD® BacLight™ are shown. The scale bar is 2 µm. The insets show a 6× magnified region from the same sample and the scale bar is 1 µm. Three representative images from three independent experiments are shown (n = 3).

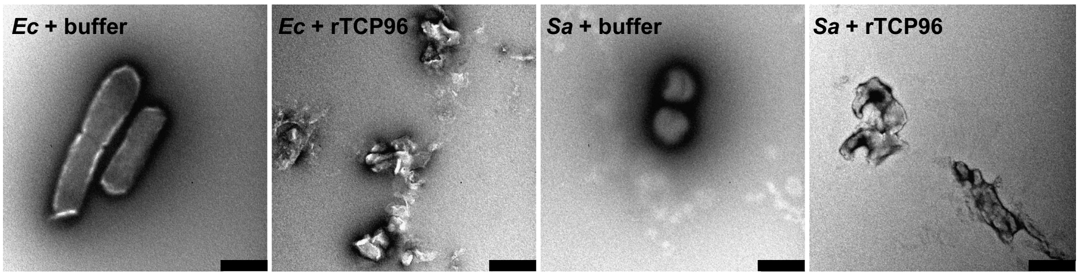

Figure S2. Transmission Electron Microscopy (TEM) analysis of bacterial viability. TEM negative staining show cell-damaging effects of rTCP96 (5 µM) on *S. aureus* ATCC 29213 (Sa) and *E. coli* ATCC 25922 (Ec). One representative image from three independent experiments is shown (n = 3). The scale bar is 1 µm.

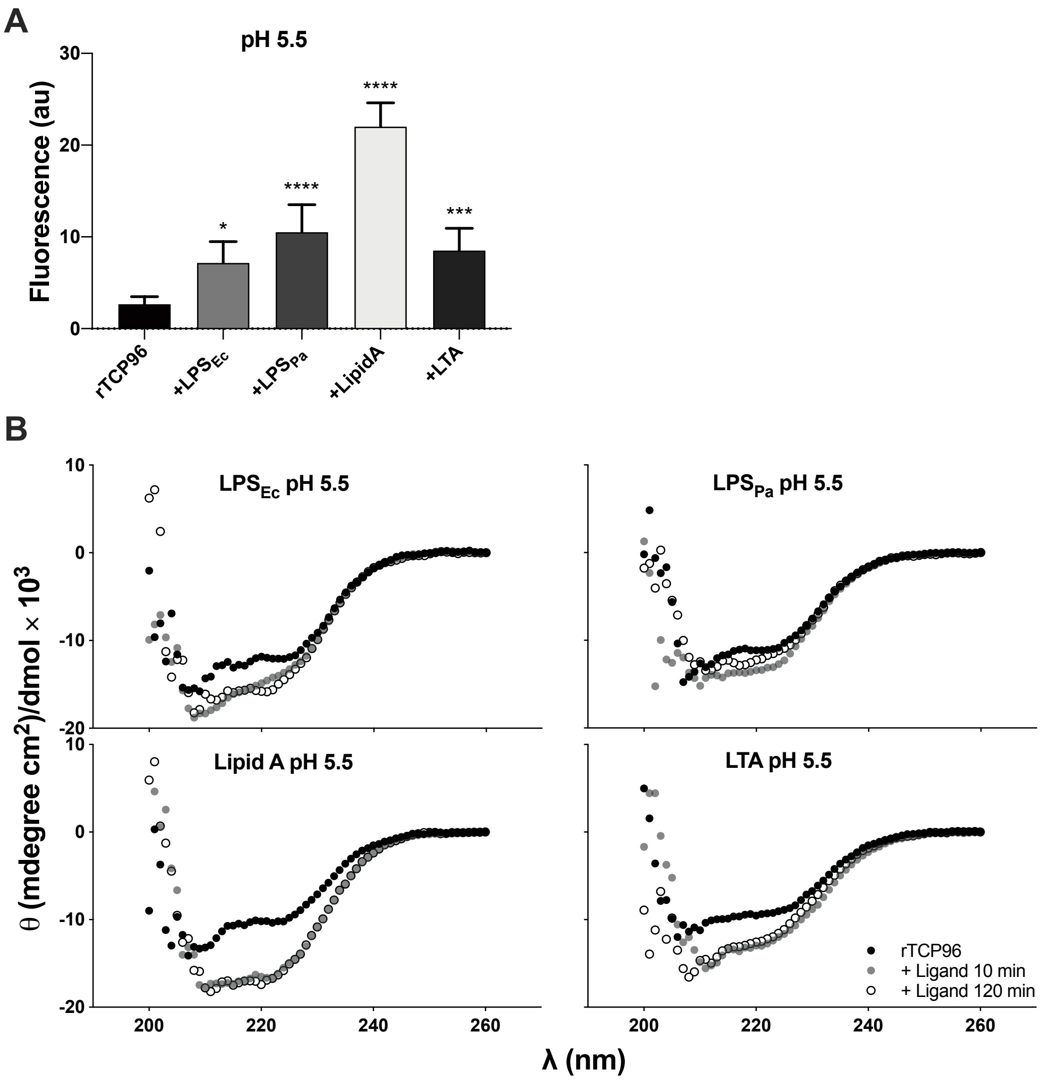

Figure S3. Aggregation and structural changes of rTCP96 in MES pH 5.5. A) ThT assay demonstrating aggregation of rTCP96 in the presence of LPS (*E. coli* and *P. aeruginosa*), Lipid A (*E. coli*) and LTA (*S. aureus*). Mean values of 3 experiments each carried out in triplicate ± their standard deviations are shown. * $P \leq 0.05$; ***$P \leq 0.005$; ****$P \leq 0.0001$. *P* values were determined using one-way ANOVA with Dunnett's multiple comparison tests (n = 6). B) Circular dichroism was used to detect an increase of β-sheet structures in rTCP96 triggered by the TLR ligands LPS (*E. coli* and *P. aeruginosa*), Lipid A (*E. coli*), and LTA (*S. aureus*)) (n = 3).

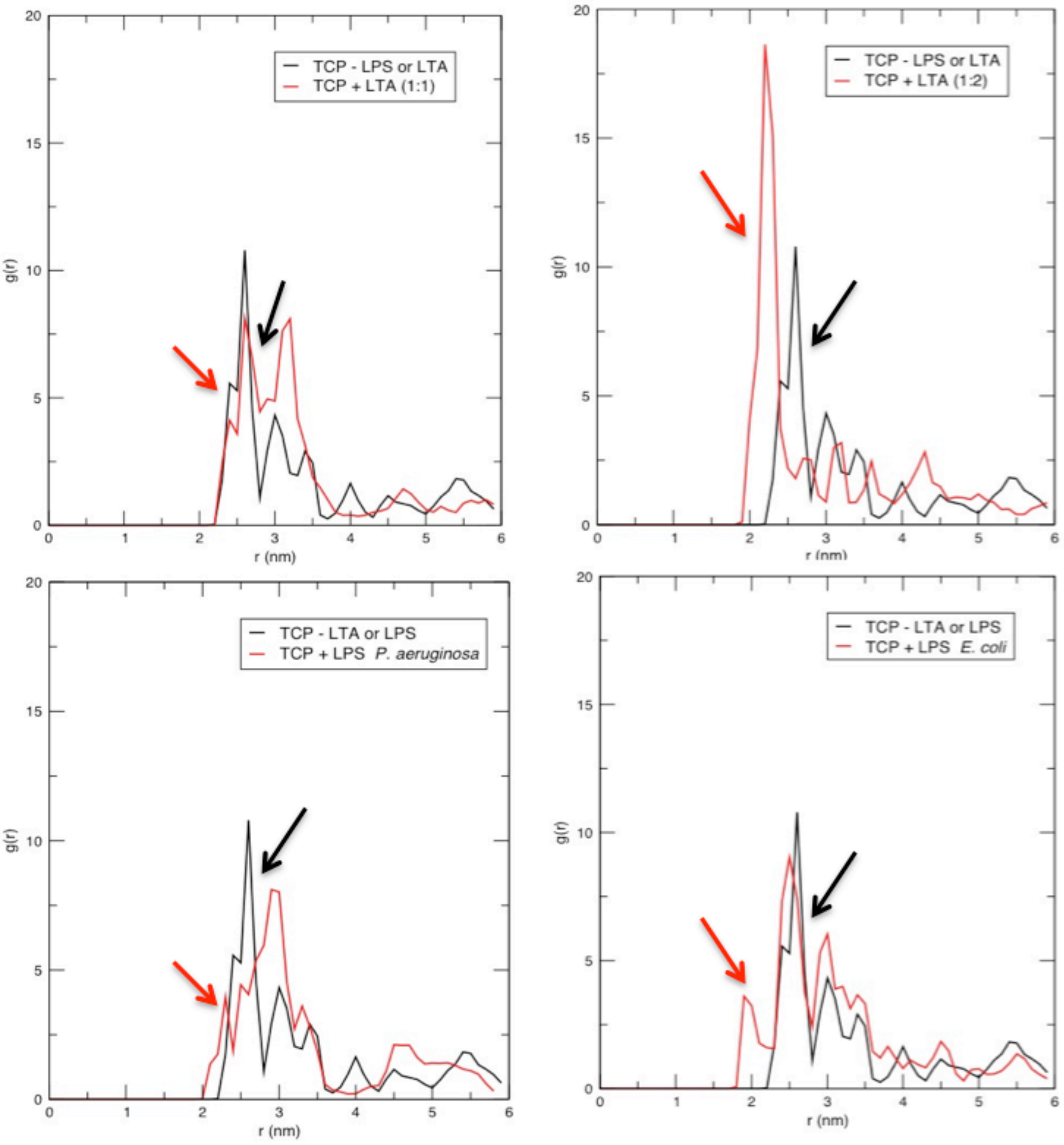

Figure S4. RDF of TCP96–TCP96 interactions for aggregates in presence/absence of different microbial products. The probability - *g(r)* - of TCP96-TCP96 interaction distances – *r* – show that in all cases TCP96 interacts more closely with other TCP96 molecules in the presence of LTA or LPS compared to in the absence of any microbial product. RDFs were calculated over 5 x1 μs simulations in the presence (red) and absence (black) of LTA/LPS. Arrows inset indicate the value taken as the first peak for determining the averages in Table 1, with black arrows presenting the TCP96–LTA/LPS lines and red representing the TCP96+LTA/LPS systems.

Simulation system	Probable average separation between TCPs in complex (nm)	P value
TCP – LTA or LPS	2.39 ± 0.015	N/A
TCP + LTA (1:1)	2.32 ± 0.015	0.01
TCP + LTA (1:2)	1.99 ± 0.038	0.0002
TCP + *P.aeruginosa* LPS	2.15 ± 0.063	0.004
TCP + *E.coli* LPS	1.97 ± 0.052	0.0007

Table S1. Separation between pairs of TCP96 fragments when in complex with different microbial products. The probable mean TCP96-TCP96 separation throughout each simulation is indicated, with standard deviations and p-values determined using a paired t-test. The separation is estimated from the first peak distance (indicated by arrows in Figure 3), from each 0.2 μs block of 1 μs simulation, after discarding the first block for equilibration purposes.
